# The applicability of non-invasive methods for assessing liver fibrosis in hemodialysis patients with chronic hepatitis C

**DOI:** 10.1371/journal.pone.0242601

**Published:** 2020-11-20

**Authors:** Jia-Jung Lee, Yu-Ju Wei, Ming-Yen Lin, Sheng-Wen Niu, Po-Yao Hsu, Jiun-Chi Huang, Tyng-Yuan Jang, Ming-Lun Yeh, Ching-I Huang, Po-Cheng Liang, Yi-Hung Lin, Ming-Yen Hsieh, Meng-Hsuan Hsieh, Szu-Chia Chen, Chia-Yen Dai, Zu-Yau Lin, Shinn-Cherng Chen, Jee-Fu Huang, Jer-Ming Chang, Shang-Jyh Hwang, Chung-Feng Huang, Yi-Wen Chiu, Wan-Long Chuang, Ming-Lung Yu

**Affiliations:** 1 Nephrology Division, Department of Internal Medicine, Kaohsiung Medical University Hospital, Kaohsiung Medical University, Kaohsiung, Taiwan; 2 Faculty of Internal Medicine, Kaohsiung Medical University, Kaohsiung, Taiwan; 3 Hepatobiliary Division, Department of Internal Medicine, Kaohsiung Medical University Hospital, Kaohsiung Medical University, Kaohsiung, Taiwan; 4 Hepatitis Research Center, Kaohsiung Medical University, Kaohsiung, Taiwan; 5 Lipid Science and Aging Research Center, College of Medicine, Kaohsiung Medical University, Kaohsiung, Taiwan; 6 Center for Cohort Study, Kaohsiung Medical University, Kaohsiung, Taiwan; 7 Center for Cancer Research, Kaohsiung Medical University, Kaohsiung, Taiwan; Nihon University School of Medicine, JAPAN

## Abstract

**Background:**

The accurate assessment of liver fibrosis among hemodialysis patients with chronic hepatitis C (CHC) is important for both treatment and for follow up strategies. Applying the non-invasive methods in general population with viral hepatitis have been successful but the applicability of the aminotransferase/platelet ratio index (APRI) or the fibrosis-4 index (FIB-4) in hemodialysis patients need further evaluation.

**Materials and methods:**

We conducted a prospective, multi-center, uremic cohort to verify the applicability of APRI and FIB-4 in identifying liver fibrosis by reference with the standard transient elastography (TE) measures.

**Results:**

There were 116 CHC cases with valid TE were enrolled in our analysis. 46 cases (39.6%) were classified as F1, 35 cases (30.2%) as F2, 11 cases (9.5%) as F3, and 24 cases (20.7%) as F4, respectively. The traditional APRI and FIB-4 criteria did not correctly identify liver fibrosis. The optimal cut-off value of APRI was 0.28 and of FIB-4 was 1.91 to best excluding liver cirrhosis with AUC of 76% and 77%, respectively. The subgroup analysis showed that female CHC hemodialysis patients had better diagnostic accuracy with 74.1% by APRI. And CHC hemodialysis patients without hypertension had better diagnostic accuracy with 78.6% by FIB-4.

**Conclusions:**

This study confirmed the traditional category level of APRI and FIB-4 were unable to identify liver fibrosis of CHC hemodialysis patients. With the adjusted cut-off value, APRI and FIB-4 still showed suboptimal diagnostic accuracy. Our results suggest the necessary of TE measures for liver fibrosis in the CHC uremic population.

## Introduction

High prevalence and increased mortality and morbidity of hemodialysis patients with viral hepatitis is an important medical issue [1–3]. Although with blood product screening and implementation of universal precaution, hemodialysis patients for their therapeutic needs of repeating vascular puncture are at risk of infection. The small but substantial annual incidence of viral hepatitis seroconversion in hemodialysis units were reported to be 0.22–6.20% of hepatitis C virus (HCV), varies by different geographic distributions [4,5]. Chronic HCV infection is associated with chronic kidney diseases and end stage renal disease (ESRD) [6,7]. Therefore, HCV is not only hyperendemic in general population of Taiwan (around 3%) [8,9], but also hyperendemic in Taiwanese uremic patients under maintenance hemodialysis (17%-34%) [10–12]. Recently, with the advance in safe and effective interferon-free antiviral therapies for HCV, the success in treatment improved patient survival, decreased risk of cancer, and were cost-saving for end stage liver failure [13,14].

Accurate assessment of liver fibrosis is mandatory because the treatment algorithm of antiviral therapy for HCV are mainly based on degree of hepatic fibrosis [15–17]. Patients with advanced fibrosis are at high risk of hepatocellular carcinoma, even under HBV suppression or HCV eradication [18,19]. Furthermore, candidates of kidney transplantation with chronic hepatitis need assessment of liver fibrosis with or without portal hypertension to decide Liver-kidney transplantation or kidney transplantation alone [18,20]. In the present era, chronic hepatitis C (CHC) patients are recommended for direct-acting antivirals (DAA) treatment for all disease stages [21]. The more liberal therapeutic indication and the importance of liver fibrosis affecting long term outcome have changed the traditional gold standard, percutaneous liver biopsy [22], to non-invasive methods for assessing the liver fibrosis [23]. Among the non-invasive methods, the transient elastography (TE) such as Fibroscan is comparable and has superior accuracy in compare with aminotransferase/platelet ratio index (APRI) and fibrosis-4 index (FIB-4) in general population with CHC [21,24–27]. In a resource-limited setting, WHO 2018 and 2016 HCV guideline gave a conditional recommendation that the APRI or FIB-4 to be used for the assessment of hepatic fibrosis [21,24]. However, the applicability of APRI or FIB-4 in hemodialysis population encountered difficulty due to their relative lower serum level of aminotransferase [28–30].

Taiwan have the world highest prevalence and incidence of end-stage renal disease needing dialysis. Our national annual incidence of HCV seroconversion in hemodialysis units were decreasing in recent years with the recent rate of 0.42% [31]. In this study, we performed the standard TE in the large, multi-center, prospective, hemodialysis cohort, the FORMOSA-LIKE group [32,33]. We aim to evaluate the applicability and to identify the cut-off-value of APRI, FIB-4 for assessing liver fibrosis status of the CHC hemodialysis patients.

## Materials and methods

### Study design and participants

The project was reviewed and approved by the ethics committee of Kaohsiung Medical University Hospital (KMUH-IRB-20130034). All participants provided their written informed consent at enrollment. The FORMOSA-LIKE group was established in 2013 included 15 hemodialysis units (one medical center, three region al core hospitals, and eleven regional clinics), and extended to 22 units in 2019 with enrolled adult uremic patients under maintenance hemodialysis from 1,680 to 2,326 [32,33]. All participants had provided informed consents and received full demographic recording and biochemistry and viral hepatitis examination.

### Measurements and definitions

All of the blood samples were collected before beginning the process of hemodialysis. Anti-HCV was determined by a third-generation enzyme immunoassay (Abbott Laboratories, North Chicago, IL). HCV RNA was measured by a real-time polymerase chain reaction assay (RealTime HCV; Abbott Molecular, Des Plaines IL, USA; detection limit: 12 IU/ml) [34]. In this study, we defined the CHC group based on HCV viremia. We also excluded HBV and HCV coinfected cases in this study to identify the non-confounded effect from different viral hepatitis.

The APRI was calculated as alanine aminotransferase (ALT)/upper limit of normal (ULN) x 100/platelet count (10^9^/L). The FIB-4 was calculated as age (years) x aspartate aminotransferase (AST) (IU/L)/platelet count (10^9^/L) x [ALT (IU/L)^1/2^]. The TE (Fibroscan, Echosens, Paris, France; detection range: 2.5 to 75 kilopascal (kPa)) were performed by a qualified and experienced operator following standard procedure. The results of TE were expressed in kPa with a median value of at least 10 valid measurements and a successful rate of more than 60%. TE failure was defined as zero valid measurement, and unreliable examinations were defined as less than 10 valid measurements, a successful rate of less than 60%, or the interquartile range (IQR) more than 30% of the median TE value [30,35].

In this study, we used the TE as the references and defined the estimated METAVIR stage by F1 as TE <7.0 kPa, F2 as TE 7.0–9.4 kPa, F3 as TE 9.5–12 kPa, and F4 as TE >12 kPa, respectively. Significant fibrosis and cirrhosis was defined as METAVIR ≥F2 and METAVIR F4, respectively [21,24,25].

### Statistical analysis

The baseline characteristics were summarized as mean ± standard deviation for continuous variables with normal distribution, and the between group differences were tested by independent T test. The continuous variables with non-normal distribution were summarized as median and interquartile range, and was tested by Mann-Whitney test. Frequencies and percentages were summarized for categorical variables. Differences between groups were compared using chi-square test for categorical variables.

We displayed the correlation of APRI and FIB-4 with TE measurements by scatter plot with loess, regression lines, and a 95% prediction region. The significance was analyzed using Spearmen’s rank correlation test. We also exhibited the distributions of APRI and FIB-4 by TE defined fibrosis categories using box plot and tested linear trends by Jonckheere-Terpstra test. To evaluate the diagnostic power, we calculated the area under the curve (AUC) using receiver operating characteristic (ROC) analysis. An attempt was made to derive a suitable clinical cut-off that would best predict the liver fibrosis status. The cut-off value was determined by choosing the point on the ROC curve with the closest distance to the point of (0,1). A patient was assessed as positive or negative according to whether the noninvasive marker value was greater than, less than, or equal to a given cut-off value. The statistical analyses were performed using the SPSS 12.0 statistical package (SPSS, Chicago, IL, USA) and SAS 9.4. (SAS Institute Inc., Cary, NC, USA). All statistical analyses were based on two-sided hypothesis tests with a significance level of p <0.05.

## Results

### Baseline characteristics of the CHC hemodialysis patients

A total of 165 HCV viremia hemodialysis patients were enrolled in this study. The mean age was 65.6 ± 13.0 years old and 87 (52.7%) of cases were males. The mean body mass index (BMI) was 22.6 ± 4.1 kilogram/m^2^. 96 (58.2%) cases had diabetes mellitus, and 111 (67.3%) of cases had hypertension. The median and interquartile range of AST level was 24 (19–32) IU/L, ALT was 22 (16–35) IU/L, and the platelet count was 168 (126–210) x 10^9^/L. The HCV RNA reads were 6.48 (4.07–7.94) Log IU/mL. The genotype analysis showed 73 cases were HCV genotype 1, 82 cases were genotype 2, and 10 cases were genotype 6. Among the study group, 116 cases had completed the TE measures. There was no differences in age, sex, underlaying disease, and laboratory parameters of the overall CHC hemodialysis group and of those with TE reads **Table 1**. The median and interquartile of TE reads was 7.8 (6.1–11.6) kPa. We further categorized our participants for liver fibrosis using the TE definition and resulted in 46 (39.6%) cases were F1, 35 (30.2%) cases were F2, 11 (9.5%) cases were F3, and 24 (20.7%) were F4 **Table 1**. In these 116 case with TE, the median and interquartile of the calculated APRI and FIB-4 were 0.34 (0.22–0.56) and 1.77 (1.31–3.28), respectively.

**Table 1:** Baseline characteristics of the hemodialysis patients with chronic hepatitis C.

	Overall	With complete TE records	P value
N	165	116	
Age (years)	65.6 (9.8)	65.4 (9.4)	0.86
Male sex	87 (52.7%)	62 (53.5%)	0.91
Pre-HD Body weight (kilograms)	60.9 (13.0)	61.2 (12.9)	0.85
Post-HD Body weight (kilograms)	58.6 (12.5)	58.8 (12.4)	0.90
BMI, kilogram/m^2^	22.6 (4.1)	22.7 (4.2)	0.78
>30	5 (3.0%)	4 (3.5%)	0.84
Diabetes Mellitus	96 (58.2%)	66 (56.9%)	0.83
Hypertension	111 (67.3%)	74 (63.8%)	0.54
AST (International unit/Liter)	24 (19–32)	23 (17–28)	0.26
ALT (International unit/Liter)	22 (16–35)	21 (15–31)	0.24
PLT (10^9^/Liter)	168 (126–210)	166 (122–212)	0.83
APRI	0.36 (0.23–0.59)	0.34 (0.22–0.56)	0.45
FIB-4	1.87 (1.35–3.00)	1.77 (1.31–3.28)	0.80
HCV RNA, (Log IU/mL)	6.48 (4.07–7.94)	6.45 (4.07–8.04)	1.00
HCV genotype 1/2/6	73/82/10	49/61/6	0.87
**Transient Elastography (kPa)**		7.8 (6.1–11.6)	
<7		46 (39.6%)	
7–9.5		35 (30.2%)	
9.5–12		11 (9.5%)	
>12		24 (20.7%)	

Abbreviations: TE, transient elastography; HD, hemodialysis; BMI, body mass index; AST, aspartate aminotransferase; ALT, alanine aminotransferase; PLT, platelet; APRI, aminotransferase/platelet ratio index; FIB-4, fibrosis-4 index.

Notes: The continuous variables with normal distribution were summarized with mean and standard deviation, and were analyzed by independent T test. The continuous variables with non-normal distribution were summarized with median and interquartile range, and were tested by Mann-Whitney test. The categorical variables were expressed with frequencies and percentages and tested the distribution difference by *X*^2^.

### Correlation between APRI, FIB-4, and the TE measurements

The scatter plots of APRI and TE in **S1 Fig**, and of FIB-4 and TE in **S2 Fig**, showed nearly linear relationship. Similarly, the raised mean values of APRI and FIB-4 with categoric TE measurements in **S3 and S4 Figs.** (P for linear trend: <0.001) suggested APRI or FIB-4 could be applied for classification of severity of liver fibrosis and cirrhosis in dialysis patients with HCV infection. The Spearman’s correlation demonstrated a significant, positive, but mild to moderate correlation between APRI, FIB-4, and TE measurements, respectively, in **Table 2**.

**Table 2:** Correlation of APRI, FIB-4, and Transient elastography methods in chronic hepatitis C dialysis patients.

	APRI	FIB-4	Transient Elastography
APRI	1.00	0.87*	0.42*
FIB-4	0.87*	1.00	0.43*
Transient Elastography	0.42*	0.43*	1.00

Data represented Spearman correlation coefficient for patients with chronic hepatitis C.

* p<0.001 by Spearman correlation test.

Abbreviation: APRI, aminotransferase/platelet ratio index; FIB-4, fibrosis-4 index.

### The diagnostic accuracy of liver fibrosis using APRI and FIB-4 in CHC hemodialysis patients

**Fig 1:**
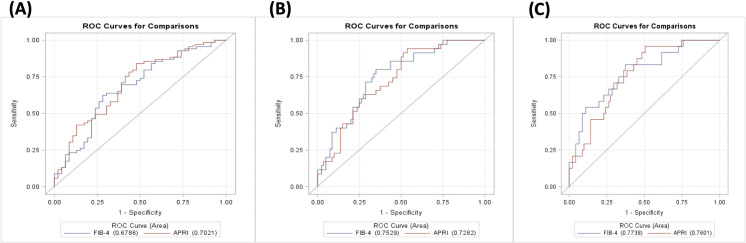
The operating characteristic curves (ROC) of APRI and FIB-4 for the prediction of patients with significant hepatic fibrosis (≥F2), advanced hepatic fibrosis (≥F3), and cirrhosis (F4) in dialysis patient with chronic hepatitis C. (A) AUC of APRI (0.70, 95% CI: 0.60 to 0.80; P = 0.004) and that of FIB-4 (0.68, 95% CI: 0.58 to 0.78; P = 0.01) for patients with a fibrosis stage of F2 or more. (B) AUC of APRI (0.73, 95% CI: 0.63 to 0.82; P = 0.002) and that of FIB-4 (0.75, 95% CI: 0.66 to 0.85; P value < 0.001) for patient with a fibrosis stage of F3 or more. (C) AUC of APRI (0.76, 95% CI: 0.66 to 0.86; P<0.001) and that of FIB-4 (0.77, 95% CI: 0.67 to 0.88; P value < 0.001) patients with a fibrosis stage of F4.

The diagnostic accuracy of APRI and FIB-4 to predict CHC hemodialysis patients with significant hepatic fibrosis (≥ F2), advanced hepatic fibrosis (≥ F3), and cirrhosis (F4) referenced by TE method were analyzed by ROC curve **Fig 1**. The selected cut-off values of APRI to predict patients with significant hepatic fibrosis, advanced hepatic fibrosis, or cirrhosis were 0.24, 0.25, and 0.28, respectively. The AUC of APRI for fibrosis stage ≥ F2, ≥ F3, and F4 in CHC dialysis patients were 0.70, 0.73, 0.76, respectively. The negative predicted value (NPV) of APRI for fibrosis stage ≥ F2, ≥ F3, and F4 in CHC dialysis patients were 68.6%, 94.9%, and 97.8%, respectively. The selected cut-off values of FIB-4 to predict patients with significant hepatic fibrosis, advanced hepatic fibrosis, or cirrhosis were 1.89, 1.89, and 1.91, respectively. The AUC of FIB-4 for fibrosis stage ≥ F2, ≥ F3, and F4 were 0.68, 0.75, 0.77, respectively. The NPV of FIB-4 for fibrosis stage ≥ F2, ≥ F3, and F4 in CHC dialysis patients were 55.9%, 88.1%, and 93.4%, respectively **Table 3**.

**Table 3:** Accuracy of APRI and FIB-4 in predicting liver fibrosis in chronic hepatitis C patients with maintenance hemodialysis.

	APRI										
	AUROC	P value	Cut-off	Mild disease, n (%)	Advanced disease, n (%)	P value	SEN, %	SPE, %	PPV, %	NPV, %	Accuracy, %
F01 vs F2-4	0.70	0.004	0.24	22 (47.8)	57 (81.4)	<0.001	84.1	52.2	72.5	68.6	71.3
F0-2 vs F3-4	0.73	0.002	0.25	43 (53.1)	33 (94.3)	<0.001	94.3	46.3	43.4	94.9	60.9
F0-3 vs F4	0.76	<0.001	0.28	46 (50.0)	22 (91.7)	<0.001	95.8	49.5	33.3	97.8	59.1
**Traditional cut-off**											
F0-1 vs F2-4	-	-	1.5	0 (0)	1 (1.4)	-	-	-	-	-	-
F4	-	-	2.0	0 (0)	1 (4.2)	-	-	-	-	-	-
	**FIB-4**										
F0-1 vs F2-4	0.68	0.01	1.89	13 (28.3)	43 (61.4)	<0.001	62.3	71.7	76.8	55.9	66.1
F0-2 vs F3-4	0.75	<0.001	1.89	28 (34.6)	27 (77.1)	<0.001	80.0	65.0	50.0	88.1	69.6
F0-3 vs F4	0.77	<0.001	1.91	34 (37.0)	20 (83.3)	<0.001	83.3	62.6	37.0	93.4	67.0
**Traditional cut-off**											
F0-1 vs F2-4	-	-	3.25	8 (17.4)	21 (30.0)	0.13	30.0	82.6	72.4	43.7	50.9
F0-3 vs F4	-	-	6.5	0 (0)	2 (8.3)	-	-	-	-	-	-

Abbreviation: APRI, aminotransferase/platelet ratio index; FIB-4, fibrosis-4 index; vs, versus; AUROC, area under receiver operating characteristic; SEN, sensitivity; SPE, specificity; PPV, positive predicted value; NPV, negative predicted value.

# represented that was tested by Fisher’s Exact Test.

The traditional cut-off value of APRI and FIB-4 in WHO guidelines were tested to predict liver fibrosis [24]. In the CHC group, the standard APRI cut-off value 1.5 for liver fibrosis and 2 for cirrhosis, respectively, identified one case (1.4%) for liver fibrosis and one case (4.2%) for cirrhosis. The standard FIB-4 cut-off value 3.25 for liver fibrosis and 6.5 for cirrhosis identified 21 (30%) cases for liver fibrosis and no cases for cirrhosis, respectively **Table 3**.

### Factors associated with the APRI and FIB-4 on the severity of liver fibrosis

We further identified possible factors that affected the severity of liver fibrosis by backward regression model regarding to APRI and FIB-4, respectively. The analysis showed that gender affected on staging of liver fibrosis by APRI. Underlaying disease with hypertension affected on staging of liver fibrosis by FIB-4 **Table 4**.

**Table 4:** Factors associated with severity of liver fibrosis.

	Odds ratio of F2-4	Odds ratio of F3-4	Odds ratio of F4
APRI	16.18 (2.41–108.55)	-	-
FIB-4	-	1.82 (1.32–2.52)	1.97 (1.39–2.79)
Sex			
Female	1.00 (Reference)	-	-
Male	2.27 (1.02–5.04)	-	-
Hypertension			
No	-	1.00 (Reference)	-
Yes	-	0.40 (0.16–0.99)	-

Abbreviation: APRI, aminotransferase/platelet ratio index; FIB-4, fibrosis-4 index; vs, versus. Age, sex, diabetes, hypertension, pre-, post-HD Body weight, FIB-4, APRI, and HCV_RNA level in log scale were included in the multiple logistic regression model.

Only preserved variables were listed after backward model selection.

By stratification, female CHC hemodialysis patients had better APRI prediction accuracy for liver fibrosis with AUC of 0.76 than male cases with AUC of 0.64, see **Table 5**. Regarding FIB-4, CHC hemodialysis patient without hypertension had better FIB-4 prediction accuracy for liver fibrosis with AUC of 0.82 than cases with hypertension with AUC of 0.72, see **Table 6**. The best cut-off value of FIB-4 in CHC hemodialysis without hypertension group was 0.32, **Table 6**, which had sensitivity of 82.4%, specificity of 76%, and accuracy 78.6%.

**Table 5:** Accuracy of APRI in predicting liver fibrosis by sex in chronic hepatitis C patients with maintenance hemodialysis.

	Male										
	AUROC	P value	Cut-off	Mild disease, n (%)	Advanced disease, n (%)	P value	SEN, %	SPE, %	PPV, %	NPV, %	Accuracy, %
F01 vs F2-4	0.64	0.09	0.75	38 (69.1)	7 (100.0)	0.17	40.5	89.5	89.5	40.5	55.7
	Female										
F01 vs F2-4	0.76	0.02	0.37	9 (31.0)	25 (100)	<0.001	88.9	59.3	68.6	84.2	74.1

Abbreviation: APRI, aminotransferase/platelet ratio index; FIB-4, fibrosis-4 index; vs, versus; AUROC, area under receiver operating characteristic; SEN, sensitivity; SPE, specificity; PPV, positive predicted value; NPV, negative predicted value.

**Table 6:** Accuracy of FIB-4 in predicting liver fibrosis by hypertension in chronic hepatitis C patients with maintenance hemodialysis.

	Without hypertension										
	AUROC	P value	Cut-off	Mild disease, n (%)	Advanced disease, n (%)	P value	SEN, %	SPE, %	PPV, %	NPV, %	Accuracy, %
F0-2 vs F3-4	0.82	0.004	0.32	2 (10.0)	17 (77.3)	<0.001	82.4	76.0	70.0	86.4	78.6
	Hypertension										
F0-2 vs F3-4	0.72	0.03	0.19	0 (0.0)	36 (49.3)	1.00	88.9	50.9	37.2	93.3	60.3

Abbreviation: APRI, aminotransferase/platelet ratio index; FIB-4, fibrosis-4 index; vs, versus; AUROC, area under receiver operating characteristic; SEN, sensitivity; SPE, specificity; PPV, positive predicted value; NPV, negative predicted value.

## Discussion

Our study tested the diagnostic accuracy of non-invasive methods for predicting liver fibrosis in 116 CHC cases with valid TE measurements **Table 1**. There were positive but mild to moderate correlation between the APRI, FIB-4, and the TE measurements **Table 2**. We confirmed the traditional cut-off value of APRI or FIB-4 to predict liver fibrosis in general population cannot be applied to the hemodialysis population. The identified cut-off values of APRI and FIB-4 in our study showed only limited diagnostic accuracy to liver fibrosis with the AUC less than 0.80 in most categories. After stratification, only the CHC hemodialysis cases without hypertension had acceptable AUC of 0.82 with the cut-off value of 0.32 of FIB-4 in predicting advanced liver fibrosis, see **Tables 3**–**6**.

Population with maintenance hemodialysis have higher prevalence of viral hepatitis and higher risk of liver disease-associated mortality and morbidity than the general population [1,3]. The inferior outcome persists even after kidney transplantation [36]. Recently, the therapeutic advances in antiviral therapy with high efficiency and less side effect improved the patient’s outcome and leaded to changes in the evaluation strategies toward non-invasive methods [21,24]. Non-invasive serum markers for assessment of liver fibrosis universally use AST and/or ALT. However, the levels of AST/ALT among uremic patients with chronic hepatitis on maintenance hemodialysis were only around one-third to corresponding general population [28]. Our study used the large, prospective, multi-center cohort, the FORMOSA-LIKE group, to identified the optimal cut-off value for assessing liver fibrosis in the dialysis patients.

Low AST and ALT level of patient under maintenance hemodialysis had been noted for long [3,28,29,37]. Lower viral load, higher spontaneous viral clearance rate, and less inflammatory activity and fibrosis by histology had been reported in CHC dialysis patients [29,32,38]. Possible membranous absorption and activating antiviral cytokines such as hepatic growth factor during dialysis sessions had been proposed [38]. In our study, the case number of HCV genotype 1/2/6 were 49/58/6, respectively. The high percentage of HCV genotype 1 and 2 of the FORMOSA-LIKE group was consistent with the reported distribution in Southern Taiwan [39,40]. The low HCV viral load and within normal range AST, ALT were in line with the previous observations **Table 1**.

The TE measurement which is a method based on ultrasound technology was applied as the reference to categorize the liver fibrosis stages in our study [21,24,30,37]. The TE has been validated as a comparable and more accurate non-invasive method for assessing CHC liver fibrosis in general population [24–27] an also in dialysis patients [30,37]. With the WHO suggested criteria including the APRI cut-off value 1.5 for significant liver fibrosis (METAVIR ≥F2), the APRI cut-off value 2.0 for liver cirrhosis (METAVIR F4), the FIB-4 cut-off value 3.25 for significant liver fibrosis (METAVIR ≥F2), and the FIB-4 cut-off value 6.5 for liver cirrhosis (METAVIR F4) [24,27], our results showed that these criteria of APRI and FIB-4 in general population failed to predict liver fibrosis in the dialysis cohort **Table 3**.

In the CHC hemodialysis cohort, AUC of the ROC analysis detected the optimal cut-off value of APRI was 0.28 and of FIB-4 was 1.91 to best excluding liver cirrhosis. To be noticed, these selected cut-off value were much lower than the standard criteria in the general population. Regarding the APRI criteria for cirrhosis in CHC uremia patients, our cut-off level 0.28 is also lower than the 0.95 reported by Schiavon LL’s group in 2007 and is lower than the 0.80 reported by Liu CH’s group in 2011 [20,30]. The changing uremic cohort to less severe liver disease was consistent with the recent report [13]. However, the diagnostic accuracy was suboptimal to be applicable in the clinical setting with all the AUCs were below 0.8 **Table 3**. Only FIB-4 cut-off level of 0.19 in the CHC hemodialysis cases without hypertension have AUC 0.8 to predict cases with significant liver fibrosis **Table 5**. The numerical value of accuracy represents the proportion of true positive results (both true positive and true negative) in a selected population. In addition to sensitivity and specificity, the accuracy is also determined by how common the disease in the selected population [41] Difference in study population may result in discrepancy of accuracy between different studies [30,41].

Our study supported the importance of TE measurement in dialysis CHC population. However, in a resource limited setting, defined criteria with high NPV can be helpful for exclusion in certain clinical situations. The European Association for the Study of the Liver (EASL) 2018 recommended CHC patients with advanced fibrosis (F3) and patient with cirrhosis (F4) should remain under surveillance for hepatocellular carcinoma every 6 months even after sustained virologic response (SVR) [42]. Our study identified the cut-off value of APRI at 0.25 for F3-4 had 94.9% NPV. It suggested that patients with APRI value lower than 0.25 had 94.9% to be discharged from the surveillance plan. Similarly, patients with FIB-4 value lower than 1.89 had 88.1% could be discharged from the surveillance plan **Table 3**. In the 2020 Taiwan consensus statement on the management of hepatitis C, patient infected with genotype 3, interferon-experienced, and with compensated cirrhosis (F4) need further consideration for regiment and duration adjustment [43]. High NPV for F4 could be useful to exclude patients with cirrhosis. Our study identified the cut-off value of APRI at 0.28 for F4 had 97.8% NPV, and the cut-off value of FIB-4 at 0.77 for F4 had 93.4% NPV **Table 3**.

The FORMOSA-LIKE cohort is a community-based, regular dialysis population. All participated subjects received universal viral hepatitis examination and were prepared for further DAA therapy if persist viremia. We applied the TE measurements as reference standard in our cohort based on this clinical setting to waive the substantial risk and limitation of liver biopsy [22,24,37]. Limitation remains due to the TE value was influenced by obesity, the skin-capsule distance, acute liver inflammation, liver congestion, recent meal, amyloidosis and cholestasis [24,25]. The mean BMI of our study cohort with TE measurement was 22.7 ± 4.2 kilogram/m^2^, and 4 of the 116 cases (3.5%) has BMI > 30 kilogram/m^2^. No liver biopsy and no intensive clinical assessment for esophageal varix, collateral circulation, encephalopathy…etc. of this community-based cohort were limitations of our study.

In summary, the results from the CHC hemodialysis patients confirmed the necessary for adjusting the cut-off value of APRI and FIB-4 to assess the liver fibrosis. However, poor applicability with low AUC was an universal finding except for CHC hemodialysis without hypertension. We therefore suggest to apply TE measurement for liver fibrosis assessment in CHC hemodialysis population.

## Supporting information

S1 Fig The scatter plot of the aminotransferase/platelet ratio index and the transient elastography measurements. (DOCX)

S2 Fig The scatter plot of the fibrosis-4 index and the transient elastography measurements. (DOCX)

S3 Fig Distribution of aminotransferase/platelet ratio index by fibroscan result. (DOCX)

S4 Fig Distribution of fibrosis-4 index by fibroscan result. (DOCX)
