# Supplementary material for: The applicability of non-invasive methods for assessing liver fibrosis in hemodialysis patients with chronic hepatitis C
Source: PLoS One. 2020 Nov 20;15(11):e0242601. doi: 10.1371/journal.pone.0242601 (PMC7678992; doi:10.1371/journal.pone.0242601)

S1 Fig. The scatter plot of the aminotransferase/platelet ratio index and the transient elastography measurements. The relationship was fitted with loess and regression lines. The area of ellipse reflects a prediction region that contains 95% of the study subjects.


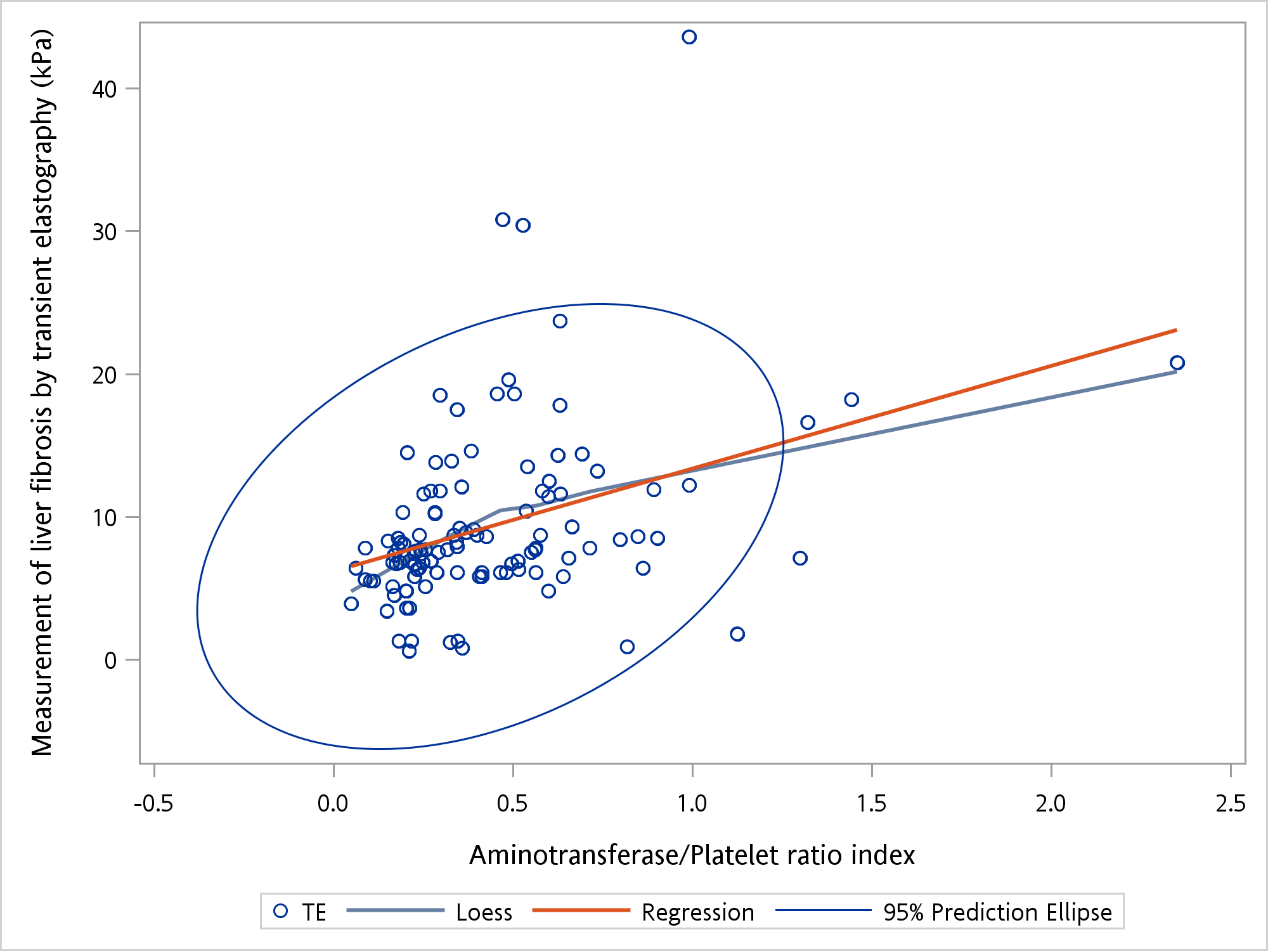

Supplement: S1 Fig — (DOCX) [file pone.0242601.s001.docx]
