# Supplementary material for: The applicability of non-invasive methods for assessing liver fibrosis in hemodialysis patients with chronic hepatitis C
Source: PLoS One. 2020 Nov 20;15(11):e0242601. doi: 10.1371/journal.pone.0242601 (PMC7678992; doi:10.1371/journal.pone.0242601)

S3 Fig. Distribution of Aminotransferase/Platelet Ratio Index by Fibroscan Result. Abbreviation: STD, standar deviation; Q1, first quartile; and Q3, third quailtile. The severity of liver fibrosis and cirrhosis was define by value of transient

elastography (TE) as F01:<7.0 , F2: 7.0-9.4, F3: TE 9.5-12, and F4: TE

>12 kPa.


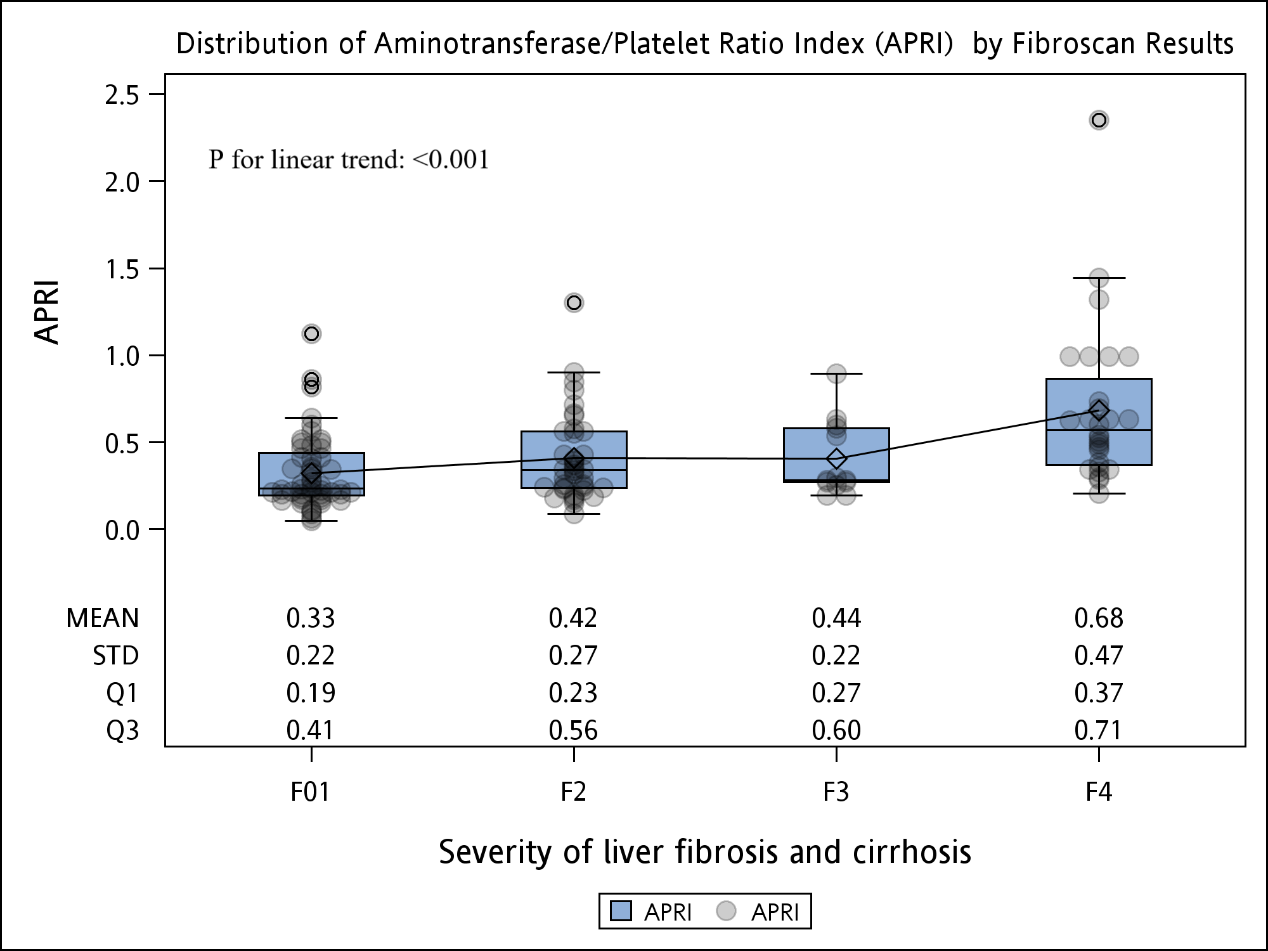

Supplement: S3 Fig — (DOCX) [file pone.0242601.s003.docx]
